# Parental imprisonment, delinquent behavior, and BMI gain in a U.S. nationally representative cohort study of adolescents and adults ages 12-32

**DOI:** 10.1016/j.ssmph.2023.101425

**Published:** 2023-05-08

**Authors:** Michael E. Roettger, Brian Houle, Jason D. Boardman

**Affiliations:** a School of Demography, 148 Ellery Crescent, The Australian National University, Acton ACT, 2601, Australia; b MRC/Wits Rural Public Health and Health Transitions Research Unit (Agincourt), School of Public Health, Faculty of Health Sciences, University of the Witwatersrand, Johannesburg, South Africa; c Institute of Behavioral Science and Department of Sociology, University of Colorado, Boulder, 1440 15th Street, Boulder, CO, 80309, USA

**Keywords:** Parental imprisonment, Delinquency, Body mass index, Social determinants of health, Life course

## Abstract

Children who experience parental imprisonment report greater mental and physical health adversities in adolescence and adulthood relative to comparable individuals whose parents did not serve time in prison. Research has linked BMI gain with parental imprisonment among females, but other studies have shown null or negative associations between parental imprisonment and weight increases for their offspring. Using longitudinal data from the National Longitudinal Study of Adolescent to Adult Health, this study attempts to resolve these differential findings by examining the interrelationship between delinquent behavior and BMI associated with parental imprisonment as individuals progress from adolescence into adulthood (ages 12–32). We show that higher delinquency levels are associated with lower BMI among men and women. With the transition from adolescence to adulthood, parental imprisonment is linked with increased BMI gain and obesity among females who are not delinquent. These findings highlight the need to consider how the decline in delinquent behavior and increasing health disparities between adolescence and adulthood may intersect as individuals experiencing parental imprisonment transition from adolescence to adulthood.

## Highlights

• We examine the relationships among BMI, delinquency, and parental imprisonment.
• Higher delinquency linked to lower BMI for those experiencing parental imprisonment.
• Parental imprisonment for non-delinquent females associated with higher BMI.
• Parental imprisonment linked with female BMI gain in early adulthood and afterwards.
• Cardiometabolic risk associated with parental imprisonment may vary by behavior-type.

## 1 Background

Paralleling the rise of imprisonment as a common life course event in the United States, children who experience parental imprisonment (PI) increasingly face economic, social, and health disparities over the life course relative to the general population (Western, 2006; Wildeman & Wang, 2017). Approximately 18% of U.S. adults, including 34% of adults aged 18–29, report that a parent has undergone imprisonment (Enns et al., 2019). Racial and ethnic minorities are particularly impacted: 24% of Black and 11% of Hispanic children cumulatively experience a parent serving time in a state or federal prison, compared with only 4% of non-Hispanic white children (Sykes & Pettit, 2014). Examining the intergenerational transmission of antisocial behavior (including delinquency) and criminal justice involvement in the context of U.S. mass imprisonment, Roettger and Dennison (2018) argued that several overlapping adversities and disadvantages associated with PI constitute a potential anthropomorphic disaster impacting health and behavior across the life course.

Over the last decade, a growing body of literature has linked PI in childhood with later-life adverse health issues. PI is linked to several adverse physical health outcomes, including migraines, respiratory illnesses, sexually transmitted infections (STIs), childhood developmental delays, physical disability, cardiovascular disease, and experiencing fair or poor health (Le et al., 2019; Lee et al., 2013; Roettger & Houle, 2021; Turney, 2014; Turney & Wildeman, 2015; Wildeman et al., 2018). Population-based studies have also linked PI with adverse birth outcomes and infant and adult mortality (Testa et al., 2019; Van De Weijer et al., 2018; Wildeman, 2012; Wildeman et al., 2014). However, data and methods limitations and a lack of knowledge about how PI is associated with adverse health among children over the life course render PI and health a critical area of study (Johnson & Easterling, 2012; Massoglia & Pridemore, 2015; Wildeman et al., 2018).

The pathways through which PI may lead to later-life adverse health outcomes and mortality are not well understood. Obesity, defined as having a body mass index (BMI) of ≥30 kg/m^2^ and recently classified as a disease by the American Medical Association, is a potential pathway because of its linkage to several health conditions, including diabetes, cardiovascular disease, hypertension, and some types of cancers. Among adults aged 20–50, obesity and BMI gain are linked with increased cardiometabolic diseases (Haffner, 2006). Meta-analysis and reviews of research on adverse childhood experiences—childhood traumas that include child abuse and neglect, parental absence, and incarceration of a family member—are childhood stressors linked with later-life cardiovascular and metabolic disease, with obesity and BMI gain as mediating mechanisms (Huang et al., 2015; Su et al., 2015; Wiss & Brewerton, 2020).

Research studies linking PI to obesity or increased BMI, however, have shown mixed results. Two studies using U.S. and Australian cohort data have shown longitudinal associations between PI and increased BMI in female respondents (Roettger & Boardman, 2012; Roettger et al., 2022). In addition, Roettger et al. (2022) reported that PI is associated with other measures of cardiometabolic risk. However, other cross-sectional studies have found either null or negative associations between PI in childhood and adulthood obesity (Branigan & Wildeman, 2019; Lee et al., 2013; Turney, 2014). One study examining the relationship between family member imprisonment (including PI) and cardiometabolic disease risk observed increased obesity and obesity-related complications among female respondents but no associations among male respondents (Lee et al., 2014). These sex-based risks are supported by one recent study linking 10.13039/100006150PI with higher C-reactive protein levels among only female adult children; high C-reactive protein levels are higher among females who are overweight or obese and influence the development of cardiometabolic diseases (Boch & Ford, 2014; Choi et al., 2013). More general research on child adversity and disadvantage is linked with increased BMI and obesity in adulthood among female populations (Khlat et al., 2009; Lee et al., 2009). The concentration of BMI gain associated with PI or familial incarceration is also consistent with research findings demonstrating that imprisonment leads to chronic health issues and health disparities among former prisoners and those who experience PI or familial imprisonment, with disproportionate effects on female and minority populations (Gjelsvik et al., 2013; Lee et al., 2014; Trotter et al., 2018; van de Weijer et al., 2021; Wildeman & Wang, 2017).

Other studies have found mixed results based on age and gender, with findings suggesting that sex and responses to stress may influence the relationship between PI and obesity/BMI gain. A large body of research has found that males are more likely to engage in externalizing behaviors (e.g., antisocial/delinquent behavior, rule breaking, etc.) in response to stress, whereas females tend to internalize stress (Smith et al., 2018). Roettger and Boardman (2012) found that obesity status mediated an association between PI and depression in female respondents, suggesting that internalization of stress at the time of measurement may explain variations in findings. However, this research did not explore whether these patterns varied for measures of externalizing behaviors, including delinquency. This omission is particularly important because although PI is linked with an increased risk of delinquency and criminal behaviors in adulthood, the prevalence of these behaviors is much lower among females (Broidy & Agnew, 1997; Kruttschnitt, 2013; Swisher & Roettger, 2012). The concentration of offending and imprisonment among families over generations is well established, potentially shaping the risk of obesity and BMI gain (Bijleveld & Wijkman, 2009; Farrington et al., 2009; Wildeman, 2020). Given the link between PI and mortality, delinquent behaviors might moderate the association between PI and weight gain by sex. However, we are unaware of studies examining the interrelationships among PI, BMI, and delinquency.

An important component of BMI gain and delinquency is their timing in the life course. Analyses examining PI using the National Longitudinal Study of Adolescent to Adult Health (Add Health) and other data sources found that delinquent behaviors (among non-chronic offenders) tend to peak in adolescence and decline in early adulthood, whereas BMI gains into overweight and obese categories more often begin in young adulthood (Fairchild et al., 2013; Moffitt et al., 2002; Roettger & Boardman, 2012; Roettger & Swisher, 2011; Yang et al., 2021). The scant research linking obesity with antisocial/delinquent behaviors has yielded mixed results (Derks et al., 2019; Mamun et al., 2009), leaving unanswered questions about the pathway between BMI gain linked to delinquent behavior and PI in childhood. **(**Roettger and Boardman's (2012) findings suggest that the association between PI and BMI might (1) occur only among females or (2) be concentrated among males or females who do not engage in externalizing behaviors such as delinquency. Given that delinquent behavior occurs most often in adolescence, BMI gain may also be more likely among those experiencing PI who have desisted from delinquency and aged into their 20s and 30s. Examining these pathways can help identify subpopulations experiencing PI who are at increased risk of cardiovascular disease due to early weight gain.

### 1.1 The current study's contributions

Using four waves of data from a cohort survey prospectively following respondents from ages 12 to 32, the current study examines delinquent behavior and BMI gain among respondents who experienced PI. Our study makes three key contributions to the literature.

First, building on prior research, we analyze how PI interacts with delinquent behavior and BMI gain over time. Several studies have analyzed the relationship between PI and delinquency/offending over time. However, as far as we are aware, only two longitudinal studies have examined PI and BMI gain, and no study has examined the longitudinal relationship between BMI gain and delinquent behavior associated with PI (Roettger & Boardman, 2012; Roettger et al., 2022; Wildeman, 2020).

Second, we examine patterns in delinquent behavior and BMI gain by age and sex for adolescents and young adults. Although multiple studies have linked BMI gain or obesity with PI/familial imprisonment, Roettger and Boardman (2012) observed this association among female respondents not experiencing depression. We expand this research by examining whether BMI gain occurs among individuals who are not delinquent and whether BMI gain occurs only among females. Further, by examining whether the association between BMI gain and delinquency is constant or emerges over time, we can establish whether the risk of BMI gain associated with PI varies with age.

Third, as Wildeman and colleagues (Wildeman et al., 2018; Wildeman & Lee, 2021) noted, longitudinal studies examining PI and physical health outcomes are rare and limited by data and statistical analysis. Time-varying measures are critical for better establishing causal inference and examining how behavioral and health risks may interrelate and evolve over time. By incorporating time-varying models in our analysis, we advance knowledge of PI and health.

## 2 Methods

### 2.1 Data

Our analysis uses four waves of the National Longitudinal Study of Adolescent to Adult Health (Add Health), a nationally representative U.S. cohort study. The study initially surveyed approximately 90,000 students enrolled in grades 7–12 (ages 12–18) in 1994–1995 during in-school interviews, from which 20,745 respondents were randomly selected and surveyed in follow-up in-home interviews. These respondents were interviewed at three later waves: 14,738 respondents at Wave 2 in 1996, 15,197 respondents at Wave 3 in 2001–2002, and 15,701 respondents at Wave 4 in 2007–2008. Of the original sample, 71%, 73%, and 75.5% completed surveys at Waves 2, 3, and 4, respectively (Harris et al., 2019). Our analytic sample comprises 15,567 individuals who completed in-home interviews at Waves 1 and 4, including 2525 Wave 4 respondents who completed a question indicating parental imprisonment for at least one biological parent (we also use the age of first parental imprisonment reported by respondents). The four waves of data allow us to examine BMI gain and experience of PI from early/late adolescence (ages 12–18) into adulthood (ages 26–32). As Wildeman et al. (2018) noted, these data are unique in allowing for longitudinal methods and analysis that can better establish causality relative to previous cross-sectional analyses with PI measures.

### 2.2 Measures

*Delinquent behavior*. Research has long documented that respondents reliably report criminal behaviors and arrests (Gomes et al., 2018; Hindelang et al., 1979). Thus, our measure of delinquency is based on respondents' self-reports on eight items in Waves 1–4 developed by Roettger et al. (2016). Items included the frequency in the prior 12 months of deliberately damaging another's property, theft under $50, theft more than $50, threatening or using a weapon to take something from someone, burglary, participating in a group fight, selling drugs, and getting into a physical fight. A higher delinquency score denotes a higher overall number of delinquent behaviors. The response options for these questions are no occurrences (= 0), one or two reported occurrences (= 1), three or four reported occurrences (= 2), and five or more occurrences (= 3). The eight items are summed, with a range of 0–24. The Cronbach's alphas for the scale are 0.76 at Wave 1, 0.74 at Wave 2, 0.68 at Wave 3, and 0.63 at Wave 4. Delinquent behavior scores are logged to reduce skewness.

*BMI (kg/m*^*2*^*)*. Height and weight were self-reported at all waves but were interviewer measured at Waves 2–4. Like Roettger and Boardman (2012), we found that Wave 2 self-reported and measured BMI are highly correlated (*r* = 0.93), suggesting that the measures are consistent during adolescence. Therefore, to obtain a longitudinal measure of BMI from Waves 1–4, we use self-reported height and weight from Wave 1 and measured height and weight from Waves 2–4. All analyses involving BMI measures from Waves 2–4 are based on measured BMI.

*Parental imprisonment*. At Wave 4, respondents were asked, “Has your biological mother/father ever been in prison?” Individuals who answered “yes,”) received a follow-up question: “How old were you when your biological mother/father went to jail or prison (the first time)?” Using these questions, we construct indicator variables for whether the biological mother/father was imprisoned. For analysis coding, we include an indicator for *any* PI and a categorical indicator for (1) mother imprisonment, (2) father imprisonment, and (3) joint mother and father imprisonment. Using age reports, we construct time-varying measures of imprisonment at each interview wave and imprisonment before age 18. Prior research suggests that recollection of childhood traumas and reporting of PI yields reliable estimates (Foster & Hagan, 2013; Winegar & Lipschitz, 1999).

The Add Health data also contain imprisonment measures for the respondent's mother and father figures, who may include stepparents, aunts, uncles, or mentors. Meta-analyses for antisocial/delinquent behavior and BMI indicate substantial heritability in families, making the combination of biological parents and parent figures problematic when examining intergenerational patterns (Burt, 2009; Elks et al., 2012). As such, we exclude parental figures from our analysis and focus on biological parents.

#### 2.2.1 Controls

*Demographic controls*. We include the respondent's age at each wave; biological sex at birth recorded at Wave 1 interviews; and whether the respondent identified as Black, white, Hispanic, Native American, Asian, or other/multiple races at Wave 1.

*Socioeconomic status*. To measure family socioeconomic status (SES), we use a scale that Ford et al. (1999) developed for Add Health data. This scale uses parent and child reports of the respondent's mother's and father's employment status and education level at Wave 1, basing family SES score on the highest mother or father's overall SES score. This measure allows us to control for the strong correlation between imprisonment and SES (Wildeman & Wang, 2017).

*Family structure.* Being raised in a single-parent household is associated with a higher risk of delinquency and obesity compared to children being raised with both biological parents (Demuth & Brown, 2004; Duriancik & Goff, 2019). We therefore use dummy measures to control for the respondent's Wave 1 familial household type: living with both biological parents, living in a household with one biological parent and one stepparent, living in a single-mother household, living in a single-father household, or living in another household type.

*Neighborhood poverty.* Neighborhood poverty rates are linked with increased obesity and delinquency rates (Hay et al., 2007; Mendez et al., 2016). Therefore, we include an indicator of the proportion of families in the respondent's census tract residing below the poverty level.

*Pregnancy*. Following Roettger and Boardman (2012), we include a measure of pregnancy to control for BMI increases associated with pregnancy. We use an indicator variable for each wave to denote whether a female was pregnant or had been pregnant in the three months before the interview.

*Difficult child temperament.* Because prior research has identified a difficult child temperament as a potential indicator of low self-control (Hagan & Foster, 2003; Roettger & Swisher, 2011), we include this variable in our analyses. At Wave 1, Add Health asked parents whether the respondent had a difficult temperament as a young child.

*School attachment*. School attachment is a three-item measure assessed via a Wave 1 question asking respondents whether they feel close to others at school, are happy at school, and feel like they are part of the school. These measures, which Roettger and Swisher (2011) also used, are associated with lower rates of delinquent behaviors.

*Birth weight*. Birth weight is a predictor of increased BMI and obesity in later life (Yu et al., 2011). This measure comes from parent reports of the child's birth weight (in pounds) at Wave 1.

*Maternal/paternal obesity*. At Wave 1 interviews, the surveyed parent was asked whether the respondent's biological mother or father is obese. Using indicators for these self-reports of the biological mother's and father's obesity allows us to control for potential increased BMI associated with biological and environmental causes of intergenerational obesity.

*Sedentary behaviors.* Given that sedentary behaviors are associated with increased BMA, and following Roettger and Boardman (2012), we include a measure for sedentary behaviors. This measure captures the average number of hours per day during the prior seven days when the respondent was sedentary, engaging in activities that included watching TV, using a computer, being on the internet, and playing video games. This measure is time-varying (assessed at Waves 1–4).

### 2.3 Analytical strategy

We use quantitative methods to explore the relationships among delinquency, PI, and BMI, examining potential variation by the respondent's biological sex and age. Given the potential complexity of these associations, we model delinquency and BMI using separate models to examine variations by outcome and sex and age.

First, we explore whether PI is associated with changes in BMI and delinquency that vary by the respondent's sex, establishing a baseline for examining whether PI is associated with sex variation in BMI and delinquency**.** Adopting the change score models that Roettger and Boardman (2012) used, we test for sex variations in changes in delinquency and BMI associated with PI between Waves 2 and 4. For BMI, our change score analysis for individual *i* is defined as follows.

(1) *Delinquency*_i,W4 – W2_ = β_0_ + β_1_*PI*_i,W4 – W2_ + β_2_*Female*_i_ + β_3_*Delinquency*_i,W2_**+ βX** + e_i_,

where *Delinquency*_i,W4 – W2_ is the change in delinquency between Waves 2 and 4, *PI*_i,W4 – W2_ is a respondent first reporting PI between Waves 2 and 4, *Female* is an indicator of whether the respondent is female, *Delinquency*_i,W2_ is the respondent's baseline delinquency level at Wave 2, **βX** is vector control variables, and e_i_ is a random disturbance term.

In a second model, we add the interaction term PI_i,W4_–_W2_ × β_3_Female_i_ for individual i such that

(2) *Delinquency*_i,W4_–_W2_ = β_0_ + β_1_*PI*_i,W4_–_W2_ + β_2_*Female*_i_ + β_4_*PI*_i,W4_–_W2_ × β_3_*Female*_i_ + β_4_*Delinquency*_i,W2_**+ βX**_i_ + e_i_.

We use this model to determine whether the respondent's sex moderates the effect of PI on the change in delinquency. Replacing the delinquency measure with BMI in equations (1), (2), we examine whether sex moderates changes in BMI.

To examine the effect of PI on delinquency and trajectories over time, we use a two-level individual growth curve model in which delinquency and BMI are modeled individually using a random disturbance term (υ_i_). For individual i at time t, the measure of delinquency is modeled as follows:

(3) *Delinquency*_it_ = β_0_ + **β**_**k**_**M**_**it**_**+ βX**_it_ + υ_i_ + ε_it_,

where *Delinquency*_it_ is the delinquency score of individual i at time t, β_0i_ is the intercept, **β**_**k**_**M**_**it**_ is the vector of variables being moderated, **βX**_**it**_ is the vector of controls, υ_i_ is the random disturbance term for individual i, and ε_it_ is random error term for individual i at time t. The **β**_**k**_**M**_**it**_ will comprise the variables being moderated to examine overall patterns in delinquency. When no terms are being interacted, **β**_**k**_**M**_**it**_ will be calculated as follows:

(4) **β**_**k**_**M**_**it**_ = β_k1_*PI*_it_ + β_k2_*Female*_i_ + β_k3_*BMI*_it_,

where *PI*_it_ is the PI status of individual i at time t, *Female*_i_ is an indicator of respondent i's sex as female, and *BMI*_it_ is the BMI of individual i at time t.

When moderation patterns of PI, sex, and BMI are interacted, equation (4) is modified to include interaction terms such that **β**_**k**_**M**_**it**_ becomes

(5) **β**_**k**_**M**_**it**_ = β_k1_*PI*_it_ + β_k2_*Female*_i_ + β_k3_*BMI*_it_+β_k4_*PI*_it_ × *Female*_i_ + β_k5_*PI*_it_ x *BMI*_ii_ + β_k6_*Female*_i_ × *BMI*_it_ + β_k7_*PI*_it_ x *Female*_i_ × *BMI*_it_.

**Fig. 1:**
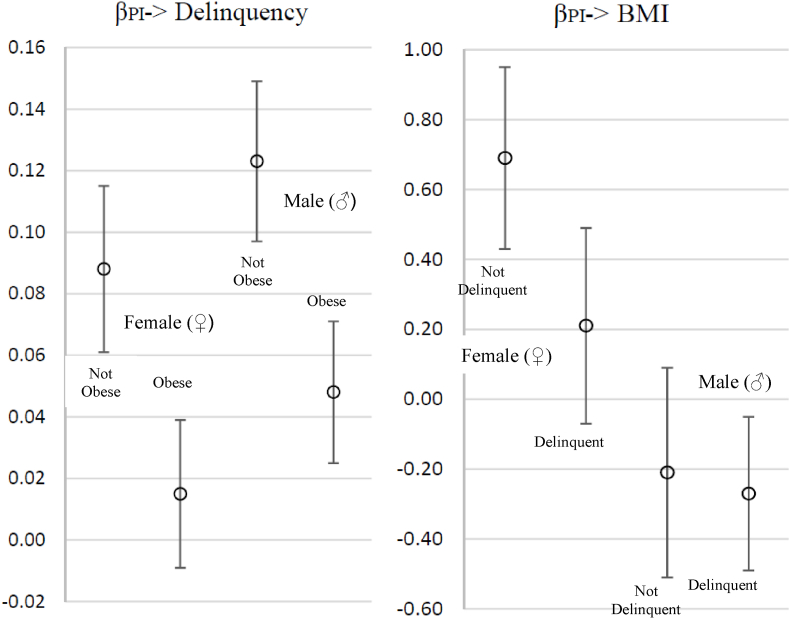
Differential effects of PI on delinquency as a function of obesity and effects of PI on the risk of obesity as a function of delinquent behaviors: The moderating role of respondent sex. Note: Estimates derived from the second and fourth models of Table 5. The points reflect the marginal effect of PI on delinquency and BMI for the left and right panels, respectively. Each panel displays four sets of estimates by respondent sex and risk status (delinquent or obese).

To aid interpretation, we plot the **β**_**k**_**M**_**it**_ interactions from equation (5) in Fig. 1.

Additionally, we examine whether the sex differences in BMI associated with PI varies by delinquency level and the respondent's age in Table 6. In doing so, we analyze results separately by respondent's sex to model a 3-way interaction for BMI of individual i at time t. Modifying equation (3), to make *BMI* the dependent variable, the equation becomes:

(6) *BMI*_it_ = β_0_ + **β**_**k**_**M**_**it**_**+ βX**_it_ + υ_i_ + ε_it_,

Equation (5) is modified to include an interaction term for PI, age, and delinquency for individual i at time t such that **β**_**k**_**M**_**it**_ becomes:

(7) **β**_**k**_**M**_**it**_ = β_k1_*PI*_it_ + β_k2_*Delinquency*_it_ + β_k3_*Age*_it_+β_k4_*PI*_it_ × *Delinquency*_it_ + β_k5_*PI*_it_ xAge_it_ + β_k6_*Delinquency*_it_ × Age_it_ + β_k7_*PI*_it_ x *Delinquency*_it_ × Age_it_.

where Age_it_ is the age of individual i at time t, while **βX**_it_, *Delinquency*_it_ and *PI*_it_ are defined previously in equations (3)–(5).

We conduct all analyses using STATA 16 (StataCorp 2019).

### 2.4 Missing data models

In all analyses, we use multiple imputation (MI) to address missing data issues. Using the STATA *ice* command, we impute 75 datasets with MI to estimate models using imputed chained equations. MI can address two data issues related to imprisonment. First, greater effects are observed for less disadvantaged respondents who have experienced PI (Turney & Wildeman, 2015), and greater disadvantage is linked with higher attrition. MI may help to reduce bias from complete cases demonstrating greater effects associated with PI. Second, in some cases, respondents reported that a parent had been imprisoned but did not know the parent's exact age at imprisonment: in 12% of cases for mothers (78/643) and 20% of cases for fathers (458/2283). We use predictive matching to estimate the missing parent's age at first imprisonment so that estimated values of age at PI match those of the non-missing data.

In all models presented in the Results section, we estimate missing data models using the controls listed earlier, as well as auxiliary variables that include the biological mother/father being unknown to the respondent, self-reported BMI for Waves 1–4, the respondent's age at their first arrest, caregiver maternal educational level, whether the respondent was foreign-born, and biological maternal/paternal closeness. We compared the results presented in this article with a complete case analysis and found no substantive variations between the imputed and non-imputed results.

## 3 Results

The distribution of PI categories from Waves 1–4 is shown in Table 1. Among those experiencing PI, approximately 90% at each wave reported paternal imprisonment. However, the percentage of respondents experiencing only maternal imprisonment and joint maternal and paternal imprisonment increased over time, cumulating to roughly 11% of respondents reporting mother-only imprisonment and 10% of respondents reporting both biological parents being imprisoned. Between Waves 1 and 4, approximately 500 respondents reported a mother or father first undergoing imprisonment.

**Table 1:** Distribution of PI by biological relationship to the incarcerated individual and wave.

Parental Imprisonment	Wave 1	Wave 2	Wave 3	Wave 4
Biological Mother	0.086	0.078	0.098	0.112
Biological Father	0.844	0.847	0.815	0.790
Biological Mother and Father	0.071	0.075	0.088	0.098
Number of Respondents	2146	1694	1957	2676
% of All Respondents in Wave	13.9%	14.4%	15.3%	17.3%

Note: All data come from Waves 1–4 of the National Longitudinal Study of Adolescent to Adult Health (Add Health). Cell entries represent proportions. The percentage of all respondents in the wave represents the proportion of all respondents completing interviews at each wave who report parental imprisonment.

Table 2 displays descriptive statistics for variables used in the analysis by the respondent's PI history. Respondents with a PI history at each wave reported significantly higher values of BMI, delinquent behavior, and sedentary behavior relative to respondents who did not experience PI during that wave. Likewise, pregnancy rates were higher among respondents with a parent imprisoned in all but Wave 4, when the average respondent was aged 28. Note that Add Health respondents' BMI increased by 6.5 units from Waves 1 to 4, while their delinquency scores declined significantly.

**Table 2:** Descriptive statistics by PI and wave.

	Wave 1			Wave 2			Wave 3			Wave 4		
	PI	No PI	*p* <	PI	No PI	*p* <	PI	No PI	*p* <	PI	No PI	*p* <
Age	15.44	15.62	0.001	16.13	16.20	0.073	21.78	21.95	0.001	28.36	28.51	0.001
Body Mass Index	22.93	22.56	0.001	23.47	23.06	0.001	26.99	26.60	0.010	29.71	29.09	0.001
Delinquent Behavior	0.72	0.52	0.001	0.55	0.41	0.001	0.33	0.26	0.001	0.21	0.11	0.001
Sedentary Behavior	3.73	3.17	0.001	3.56	2.98	0.001	3.64	3.14	0.001	2.64	2.25	0.001
Pregnant at Interview	0.03	0.02	0.001	0.04	0.02	0.001	0.05	0.04	0.004	0.06	0.05	0.800
Number of Respondents	2146	13,421		1694	10,025		1957	10,803		2676	12,891	

Note: All data come from Waves 1–4 of the National Longitudinal Study of Adolescent to Adult Health (Add Health). Cell entries represent means for continuous variables and proportions for binary variables.

Table 3 provides additional descriptive statistics for variables assessed only at one point. We find that relative to other respondents, those experiencing PI are statistically significantly more likely to be Black, less likely to be non-Hispanic white, less likely to be foreign-born, more likely to experience arrest, less likely to live with two biological parents, and have lower parental education and SES, lower school attachment, higher neighborhood poverty, and lower birth weight.

**Table 3:** Time-invariant descriptive statistics by PI status.

	PI	No PI	Pr. <
Race/Ethnicity			
White	0.465	0.542	0.001
Black	0.314	0.205	0.001
Hispanic	0.163	0.159	0.634
Asian	0.023	0.069	0.001
Native American	0.028	0.015	0.001
Other/Multiple Races	0.008	0.009	0.642
Foreign-born	0.029	0.070	0.001
Male	0.449	0.472	0.033
Years Since Last Arrest (Wave 4)			
Never Arrested	0.579	0.751	0.001
0–4	0.216	0.113	0.001
5–9	0.124	0.086	0.001
10–14	0.075	0.046	0.001
15+	0.003	0.002	0.292

Family Structure (Wave 1)			
Two Biological Parents	0.230	0.585	0.001
Single Mother	0.356	0.210	0.001
Single Father	0.047	0.031	0.001
Two Parents (one biological)	0.241	0.128	0.001
Other Family Structure	0.127	0.047	0.001
Parent Education (self-reported) (Wave 1)			
Bachelor's Degree	0.150	0.259	0.001
High School	0.607	0.579	0.007
Less Than High School	0.243	0.162	0.001
Family SES (Wave 1)	5.329	6.343	0.001
School Attachment (Wave 1)	3.650	3.760	0.001
Neighborhood Poverty (Wave 1)	0.143	0.114	0.001
Difficult Child Temperament (Wave 1)	0.381	0.293	0.001
Respondent's Birth Weight (lbs.) (Wave 1)	7.060	7.270	0.001
Parental Obesity (Wave 1)			
Maternal	0.175	0.187	0.174
Paternal	0.086	0.107	0.005
Sample Size	2676	12,891	0.005

Note: All data come from Waves 1–4 of the National Longitudinal Study of Adolescent to Adult Health (Add Health). Cell entries represent means for continuous variables and proportions for binary variables.

Table 4 provides estimates of changes in measured BMI between adolescence (Wave 2) and adulthood (Wave 4) for delinquency and BMI. For delinquency changes from Waves 2 to 4, we find in Model 1 that PI is associated with increased delinquency scores (*b* = 0.06, *p* < .01) and that females have reduced delinquency scores. Interacting PI and sex in Model 2, we observe a significant interaction (*b* = −0.05, *p* < .05): among those experiencing PI, females have lower delinquency than males. For BMI changes from Waves 2 to 4, we find no significant changes in BMI for PI and females in Model 1. However, when we interact PI and respondent's sex in Model 2, we find BMI among females experiencing PI increased by 1.34 units (*b* = 1.34, *p* < .01). These results suggest that among respondents experiencing PI, females are more likely to show lower delinquency levels and higher BMI scores relative to males.

**Table 4:** Sex differences in the effects of PI on changes in delinquency and BMI.

	Change in Delinquency Waves 2 to 4		Change in BMI Waves 2 to 4	
	Model 1	Model 2	Model 3	Model 4
BMI, Wave 2			0.10*** (0.01)	0.10*** (0.01)
Delinquency, Wave 2	−0.88*** (0.01)	−0.89*** (0.01)		
PI, Wave 2–Wave 4	0.06** (0.02)	−0.11*** (0.03)	0.17 (0.36)	−0.58 (0.33)
Female	−0.11*** (0.01)	−0.11*** (0.01)	−0.13 (0.10)	−0.16 (0.10)
PI × Female		−0.09* (0.04)		1.34** (0.51)
Constant	0.43*** (0.11)	0.42*** (0.11)	9.80*** (1.45)	9.81*** (1.45)
Sample Size	15,567	15,567	15,567	15,567

Notes: Cell entries represent regression estimates with standard errors in parentheses. For women, models exclude pregnant female respondents at Wave 2 or 4.

*p < .05; **p < .01; ***p < .001.

Table 5 presents models for Waves 1–4 estimating the effects of PI on delinquency as a function of BMI and the effects of PI on BMI as a function of delinquency among males and females separately using a three-way interaction. The results from the first model provide additional support for the findings that 10.13039/100006150PI is strongly associated with increased delinquent behavior (*p* < .001) but that females have lower levels of delinquent behavior (*p* < .001). The results from Model 3 in Table 5 validate our results from Model 3 of Table 4: PI is associated with increased BMI regardless of the wide range of statistical controls. The second and fourth models in Table 5 evaluate the key question in our paper. The second model includes a three-way interaction between PI, BMI, and respondent's sex, as well as corresponding two-way interactions as determinants of delinquency. The three-way interaction examines the possibility that the effect of PI on delinquency will be reduced among those with higher BMIs and that this interaction will differ by respondent's sex. We find support for the first question: the effect of 10.13039/100006150PI on delinquency is weaker among those with higher 10.13039/100000993BMI levels (*b* = −0.007, *p* < .001), but this effect is the same for males and females (*b* = 0.002, non-significant).

**Table 5:** Changes in delinquency and BMI as a function of PI: Sex differences in moderated effects of coping behaviors related to PI.

	Delinquency (DEL)	BMI		
	Model 1	Model 2	Model 3	Model 4
PI	0.088*** (0.010)	0.278*** (0.050)	0.199* (0.101)	−0.216 (0.151)
Female	−0.229*** (0.006)	−0.383*** (0.023)	0.02 (0.086)	−0.162 (0.094)
BMI	−0.001 (0.000)	−0.006*** (0.001)		
PI × BMI		−0.007*** (0.002)		
PI × Female		−0.088 (0.061)		
BMI × Female		0.006*** (0.001)		
PI × BMI × Female		0.002 (0.002)		
DEL			−0.01 (0.030)	−0.025 (0.152)
PI × DEL				−0.245*** (0.093)
PI × Female				0.898*** (0.203)
DEL × Female				0.151* (0.064)
PI × DEL × Female				−0.340* (0.144)

Notes: Cell entries represent regression estimates, with standard errors shown in parentheses. All models include controls for age, race/ethnicity, Wave 1 family structure, Wave 1 family SES, Wave 1 school attachment, foreign-born, Wave 1 proportion of census tracts living below the poverty level, difficult child temperament, birth weight, pregnancy status (for female respondents), sedentary behaviors, maternal obesity, and paternal obesity. Time-varying covariates are PI, deviance, age, pregnancy status, and sedentary behaviors.

*p < .05; **p < .01; ***p < .001.

**Table 6:** Changes in BMI as a function of age, delinquency (DEL), and PI, by respondent's sex.

	Males		Females	
	Model 1	Model 2	Model 3	Model 4
PI	0.021 (0.137)	0.613 (0.319)	0.488*** (0.142)	−0.559 (0.321)
DEL	−0.059 (0.035)	0.715*** (0.146)	−0.007 (0.050)	−0.914*** ((0.223)
Age	−0.492*** (0.004)	1.19 (0.033)	0.521*** (0.004)	0.51*** (0.005)
PI × DEL		−0.015 (0.323)		1.35** (0.461)
PI × Age		−0.024* (0.012)		0.051*** (0.012)
DEL × Age		−0.040*** (0.007)		0.056*** (0.012)
PI × DEL × Age		−0.007 (0.015)		−0.092*** (0.025)
Sample Size	7287	7287	8280	8280

Notes: Cell entries represent regression estimates with standard errors in parentheses. All models include controls for age, race/ethnicity, Wave 1 family structure, Wave 1 family SES, Wave 1 school attachment, foreign-born, Wave 1 proportion of the census tract living below the poverty level, difficult child temperament, birth weight, pregnancy status (for female respondents), sedentary behaviors, maternal obesity, and paternal obesity. Time-varying covariates are PI, deviance, age, pregnancy status, and sedentary behaviors.

*p < .05; **p < .01; ***p < .001.

This association is shown graphically in the left panel of Fig. 1, where the circles indicate the marginal effect of PI on delinquency among males and females by obesity status (BMI ≥30). The first two lines are for females, and the second two are for males. As mentioned earlier, an important finding of our analyses is that the effects of PI on delinquency are significantly lower for obese relative to non-obese individuals. Those who are not obese are less likely to respond to the strain of having a parent imprisoned by engaging in delinquent behaviors. However, as shown by the non-significant interaction term and the plotted values in Fig. 1, the moderating effect of obesity status is nearly identical for females and males. This is not the case when considering the moderating effect of delinquency on BMI.

The fourth model evaluates similar questions as the second model: whether the effects of PI on BMI differ for males and females and, if so, whether these sex-specific effects are moderated by delinquency levels. In this model, we show a main effect of PI on BMI (*b* = 0.196, *p* < .05), a reduced association between PI and BMI among males who are delinquent (*b* = −0.245, *p* < .001), an increased association between PI and BMI for females (*b* = 0.898, *p* < .001), and an increased effect of delinquency on BMI for females. We also find a significant decrease in the effect of PI among females with high delinquency levels (*b* = −0.340, *p* < .05). To explore these complex interactions, we also plot the marginal effects of PI for males and females by delinquency status in Fig. 1. Two important associations shown on the right side of the figure are noteworthy. First, the direction of the effects of PI on BMI for males is opposite that of females. In fact, there are no significant associations between PI and BMI for non-delinquent males and marginally protective effects among delinquent males. However, we find substantial and significant effects of PI on BMI for females who are not delinquent. These results, again, are in line with our expectations.

**Fig. 2:**
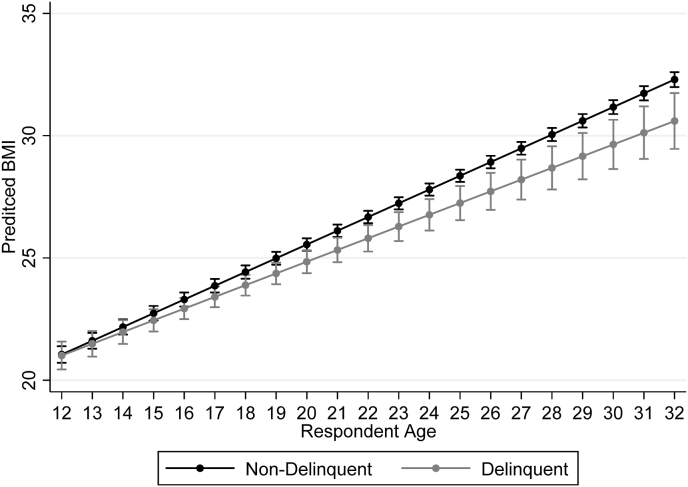
Differential effects of PI on BMI trajectories based on female respondents' age and delinquency status Note: Estimates derived from Model 4 of Table 6. The points reflect the marginal effect of PI on BMI based on female respondents being delinquent or non-delinquent.

Separating analyses by respondent sex, the interactions in Table 6 examine significant variation by PI, delinquency level, and respondent's age. For male respondents, we find no associations among PI, age, and delinquency level in Model 2. However, age, PI, and delinquency levels show a significant three-way interaction (*p* < .001) for female respondents in Model 4. Plotting these results among female respondents experiencing PI by delinquency status, Fig. 2 shows a pattern of diverging slopes in BMI as respondents age from adolescence into adulthood. In adolescence, BMI differences for delinquent and non-delinquent respondents are within 95% CI. However, BMI for non-delinquent respondents becomes significantly higher in early adulthood. This trend increases until female respondents reach their early 30s, when BMI is 1.5 units larger for non-delinquent females experiencing PI relative to delinquent females experiencing PI.

## 4 Discussion

The present study examines the relationships among BMI/obesity, delinquent behavior, and PI as individuals age from adolescence into adulthood. Our longitudinal analysis finds that higher BMI levels are linked with reduced delinquency levels. We also find sex variations in BMI gain associated with PI: female respondents who desist from or engage in low levels of delinquent behavior are more likely to experience BMI gain; however, males who experience PI and females engage in delinquency are not more likely to have increased BMI when compared to respondents not experiencing PI. This risk of relatively higher BMI associated with PI becomes more pronounced for non-delinquent females as they age from adolescence into young adulthood, a period when desistance from delinquent behaviors commonly occurs. These findings hold when we use change score models and individual growth curve models with time-varying measures of BMI, delinquency, and PI; these models show prospective changes in BMI and weight gain that better establish causality than prior research.

Our finding that higher delinquency is linked with lower BMI parallels prior research on the healthy prisoner hypothesis, which posits that imprisonment is linked with better health (Baćak & Wildeman, 2015; Houle, 2011). This overall association holds in the Add Health sample for all males and females who experienced PI in adolescence and early adulthood. Future research extending into midlife may help to better establish pathways linked to increased premature illness and mortality risk.

For females, our study provides evidence that PI and related factors contribute to early BMI gain and obesity among those not engaged in delinquency. These outcomes, in turn, may increase risks of diabetes, hypertension, and cardiovascular diseases, as Roettger et al. (2022) and Lee et al. (2014) found when examining PI and familial incarceration. The broader research literature has also linked criminal behavior, imprisonment, and PI with poor or risky health behaviors, lack of health care access, health problems, and early mortality (Heard-Garris et al., 2018; Massoglia & Pridemore, 2015; Massoglia & Remster, 2019; Semenza et al., 2020; Van De Weijer et al., 2018). The effects of PI and other childhood traumas combined with poor health behaviors, barriers to health care access, and other health risk factors may lead to cumulative disadvantages in health that culminate in premature later-life morbidity and mortality from cardiovascular and metabolic diseases.

From a life course perspective, considering the broader social context linking PI to health is also critical. One recent study explored the relationship between health trajectories and antisocial/delinquent behavior over the life course, but our findings also point to how these associations play out over time and potentially over generations in families where offending and imprisonment may be concentrated. Cumulative health disadvantages associated with PI are also part of a broad set of disadvantages associated with PI, including economic, social, educational, and criminal justice inequalities that extend across the life course and over generations (Cho, 2009; Hagan & Foster, 2015; Murray et al., 2014; Wildeman & Wang, 2017). Roettger and Dennison (2018) suggested that intergenerational offending and imprisonment may be viewed from the framework of a social disaster, with PI leading to several disadvantages that accumulate over time. As a set of life course events experienced within a disaster framework, health issues arise from a set of events, such as food insecurity, social exclusion, and substance use, which lead to increased morbidity and mortality risks as individuals progress through the life course (Cohan, 2010; DeWaard, 2016).

Life course theory provides useful insights for developing interventions and policies critical for ameliorating health disparities emerging in later life for individuals who experience PI (Jones et al., 2019). As with intergenerational offending and imprisonment, BMI and obesity are observed between parents and children, and policies promoting improved health may target both parents and children (Benyshek, 2013; Whitaker et al., 2010). As noted earlier, PI is linked with reduced health care access, increased health issues, and poorer health behaviors. Life course interventions that ameliorate these disparities may also improve health issues linked with increased BMI and obesity (Foster & Hagan, 2007; Heard-Garris et al., 2018; Lee et al., 2013). Critically, although research has demonstrated that the etiological causes of obesity and BMI gain are complex, these causes may be treated so that the effects of BMI gain and obesity associated with PI may be ameliorated over time and across generations (Benyshek, 2013; Newton et al., 2017; Pérez-Escamilla & Kac, 2013). Such strategies are critical for untangling health inequalities from the collateral consequences of mass imprisonment in American society.

The relationship between PI and delinquency is well-established in the research literature. Although the United States lacks comprehensive policies and interventions (Arditti & Johnson, 2022; Wildeman, 2020), ongoing research suggests ways to reduce the risks of delinquency and imprisonment linked to PI. Alternatives to PI for non-violent offenses are linked with reduced offending rates among children (Wildeman & Andersen, 2017). Encouraging closeness to a father figure and shifting away from deviant social networks may ameliorate delinquency associated with PI; improving health behaviors, treating substance use issues, and improving health care access may ameliorate health issues associated with PI and subsequent delinquency and criminal justice involvement (Massoglia & Remster, 2019; Roettger & Dennison, 2018; Testa & Semenza, 2020; Wildeman, 2020). With comprehensive policies and interventions addressing delinquency, health risks, and other adversities, children experiencing PI may experience substantially improved health and well-being over the life course.

### 4.1 Limitations

This study has several limitations. The Add Health data lack information on issues related to PI, such as parental criminality, substance abuse, and exposure to adverse childhood experiences (Arditti, 2015; Giordano, 2010; Wildeman & Andersen, 2017). We therefore cannot determine causality. At Wave 4, PI was measured retrospectively for adults aged 26–32. Although these reports are consistent across waves in Add Health, other research has found that official records may lead to higher imprisonment estimates at each wave (Foster & Hagan, 2013; Geller et al., 2016). Measures of child's age at PI may be less accurate when respondents were young children or the parent was frequently incarcerated. Although our analyses focus on the relationship between BMI and delinquency among individuals experiencing PI, it remains critical to consider potential relationships with other factors, such as mental and physical health. Both BMI and delinquency have been shown to have significant heritability, and our analysis does not examine potential underlying genetic and environmental factors that may explain this association (Barnes et al., 2014; Elks et al., 2012). Our results are based on a combination of prospective BMI and delinquency measures and retrospective PI measures. Studies using administrative and prospective PI measures could increase the reliability of the findings.

Also critical to consider are the limitations of the Add Health age sample. Although the cohort data capture transitions from adolescence to adulthood, the Add Health sample cannot directly link PI and other childhood adversities with several conditions that are more likely to occur in later life, such as cardiovascular disease, cancers, and stroke. Birth cohorts, administrative data, and prospective multigenerational studies can examine the potential influence of PI and associated traumatic events in childhood on later-life morbidity and mortality. This sort of examination is particularly relevant given that Wave 2 does not include respondents who graduated from high school after Wave 1 (which produced the decline in sample size in Table 1). These respondents were followed again starting in Wave 3, but readers should consider this limitation when interpreting the results of change score models using Wave 2 and Wave 4 measures of BMI. We chose to make use Wave 2 measured height and weight in our change-score models, due to Wave 1 only having self-reported height and weight. We examined differences in the overall results and found the same patterns. Still, it remains important to consider that the composition of respondents changed non-randomly between Waves 1 & 2, potentially impacting our findings.

We also urge caution in overly-generalizing these findings for delinquency to broader externalizing behaviors. Widely used scales, such as the Child Behavioral Checklist (CBCL), define externalizing behaviors to include conduct disorder, aggression, rule-breaking, and oppositional-defiant behaviors (Spatola et al., 2007). Future research may examine broader externalizing behaviors associated with parental imprisonment and their relationship to later-life health.

## 5 Conclusion

Using a US national cohort of respondents ages 12–32, the present study investigates the interrelationships between PI, BMI gain, and delinquency as individuals age from adolescence into adulthood. Our findings show that, for delinquency, higher BMI is associated with a decline in delinquency among male and female children who have experienced PI. For BMI gain, parental imprisonment is associated with a higher BMI among females who are not delinquent, when compared to delinquent females. The difference in BMI gain among delinquent and non-delinquent females experiencing PI becomes statistically significant in early adulthood, with BMI gain for non-delinquent women experiencing PI increasing with age. In contrast, for male respondents, no main or moderating effect is found between PI and BMI gain. Taken together, these findings show that increased BMI may reduce delinquency among those who have experienced PI; however, the association between BMI gain and PI among females is moderated by the level of delinquency. Non-delinquent females who have experienced PI may, as such, be at greater risk for early cardiovascular and metabolic diseases as they progress through adulthood.

## Financial interest

All authors declare no financial interests related to the publication of this mansuscript.

## Author contributions

Michael E. Roettger: Conceptualization, Methodology, Formal Analysis, Writing - Original Draft. Brian Houle: Conceptualization, Methodology, Writing - Review & Editing. Jason Boardman: Conceptualization, Methodology, Visualization, Writing - Review & Editing.

## Ethical statement

This project was reviewed and approved by The Australian National University Human Ethnics Research Committee (approval #: 2018/283).

## Declaration of competing interest

All authors declare no conflicts of interest related to publication of this manuscript.

## Data Availability

The data that has been used is confidential.

## References

[bib1] Arditti J.A. (2015). Family process perspective on the heterogeneous effects of maternal incarceration on child wellbeing. Criminology & Public Policy.

[bib2] Arditti J.A., Johnson E.I. (2022). A family resilience agenda for understanding and responding to parental incarceration. American Psychologist.

[bib3] Baćak V., Wildeman C. (2015). An empirical assessment of the “healthy prisoner hypothesis”. Social Science & Medicine.

[bib4] Barnes J.C., Wright J.P., Boutwell B.B., Schwartz J.A., Connolly E.J., Nedelec J.L., Beaver K.M. (2014). Demonstrating the validity of twin research in criminology. Criminology.

[bib5] Benyshek D.C. (2013). The “early life” origins of obesity-related health disorders: New discoveries regarding the intergenerational transmission of developmentally programmed traits in the global cardiometabolic health crisis. American Journal of Physical Anthropology.

[bib6] Bijleveld C.C., Wijkman M. (2009). Intergenerational continuity in convictions: A five‐generation study. Criminal Behaviour and Mental Health.

[bib7] Boch S.J., Ford J.L. (2014). C-reactive protein levels among U.S. Adults exposed to parental incarceration. Biological Research For Nursing.

[bib8] Branigan A.R., Wildeman C. (2019). Parental incarceration and child overweight: Results from a sample of disadvantaged children in the United States. Public Health Reports.

[bib9] Broidy L., Agnew R. (1997). Gender and crime: A general strain theory perspective. Journal of Research in Crime and Delinquency.

[bib10] Burt S.A. (2009). Are there meaningful etiological differences within antisocial behavior? Results of a meta-analysis. Clinical Psychology Review.

[bib11] Cho R.M. (2009). The impact of maternal imprisonment on children's educational achievement results from children in Chicago public schools. Journal of Human Resources.

[bib12] Choi J., Joseph L., Pilote L. (2013). Obesity and C‐reactive protein in various populations: A systematic review and meta‐analysis. Obesity Reviews.

[bib13] Cohan C.L. (2010). Handbook of stressful transitions across the lifespan.

[bib14] Demuth S., Brown S.L. (2004). Family structure, family processes, and adolescent delinquency: The significance of parental absence versus parental gender. Journal of Research in Crime and Delinquency.

[bib15] Derks I.P.M., Bolhuis K., Yalcin Z., Gaillard R., Hillegers M.H.J., Larsson H., Lundström S., Lichtenstein P., van Beijsterveldt C.E.M., Bartels M., Boomsma D.I., Tiemeier H., Jansen P.W. (2019). Testing bidirectional associations between childhood aggression and BMI: Results from three cohorts. Obesity.

[bib16] DeWaard J., Shanahan M.J., Mortimer J.T., Kirkpatrick Johnson M. (2016). Handbook of the life course: Volume II.

[bib17] Duriancik D.M., Goff C.R. (2019). Children of single-parent households are at a higher risk of obesity: A systematic review. Journal of Child Health Care.

[bib18] Elks C., Den Hoed M., Zhao J.H., Sharp S., Wareham N., Loos R., Ong K. (2012). Variability in the heritability of body mass index: A systematic review and meta-regression [original research]. Frontiers in Endocrinology.

[bib19] Enns P., Yi Y., Comfort M., Goldman A., Lee H., Muller C., Wakefield S., Wang E., Wildeman C. (2019). What percentage of Americans have ever had a family member incarcerated?: Evidence from the family history of incarceration survey (FamHIS). Socius.

[bib20] Fairchild G., van Goozen S.H.M., Calder A.J., Goodyer I.M. (2013). Research Review: Evaluating and reformulating the developmental taxonomic theory of antisocial behaviour. Journal of Child Psychology and Psychiatry.

[bib21] Farrington D.P., Coid J.W., Murray J. (2009). Family factors in the intergenerational transmission of offending. Criminal Behaviour and Mental Health.

[bib22] Ford C.A., Bearman P.S., Moody J. (1999). Foregone health care among adolescents. JAMA.

[bib23] Foster H., Hagan J. (2007). Incarceration and intergenerational social exclusion. Social Problems.

[bib24] Foster H., Hagan J. (2013). Maternal and paternal imprisonment in the stress process. Social Science Research.

[bib25] Geller A., Jaeger K., Pace G.T. (2016). Surveys, records, and the study of incarceration in families. The Annals of the American Academy of Political and Social Science.

[bib26] Giordano P.C. (2010).

[bib27] Gjelsvik A., Dumont D.M., Nunn A. (2013). Peer reviewed: Incarceration of a household member and hispanic health disparities: Childhood exposure and adult chronic disease risk behaviors. Preventing Chronic Disease.

[bib28] Gomes H.S., Maia Â., Farrington D.P. (2018). Measuring offending: Self-reports, official records, systematic observation and experimentation. Crime Psychology Review.

[bib29] Haffner S.M. (2006). Relationship of metabolic risk factors and development of cardiovascular disease and diabetes. Obesity.

[bib30] Hagan J., Foster H. (2003). S/he's a rebel: Toward a sequential stress theory of delinquency and gendered pathways to disadvantage in emerging adulthood. Social Forces.

[bib31] Hagan J., Foster H. (2015). Mass incarceration, parental imprisonment, and the great recession: Intergenerational sources of severe deprivation in America. RSF: The Russell Sage Foundation Journal of the Social Sciences.

[bib32] Harris K.M., Halpern C.T., Whitsel E.A., Hussey J.M., Killeya-Jones L.A., Tabor J., Dean S.C. (2019). Cohort profile: The national longitudinal study of adolescent to adult health (add health). International Journal of Epidemiology.

[bib33] Hay C., Fortson E.N., Hollist D.R., Altheimer I., Schaible L.M. (2007). Compounded risk: The implications for delinquency of coming from a poor family that lives in a poor community. Journal of Youth and Adolescence.

[bib34] Heard-Garris N., Winkelman T.N.A., Choi H., Miller A.K., Kan K., Shlafer R., Davis M.M. (2018). Health care use and health behaviors among young adults with history of parental incarceration. Pediatrics.

[bib35] Hindelang M.J., Hirschi T., Weis J.G. (1979). Correlates of delinquency: The illusion of discrepancy between self-report and official measures. American Sociological Review.

[bib36] Houle B. (2011). Obesity disparities among disadvantaged men: National adult male inmate prevalence pooled with non-incarcerated estimates, United States, 2002–2004. Social Science & Medicine.

[bib37] Huang H., Yan P., Shan Z., Chen S., Li M., Luo C., Gao H., Hao L., Liu L. (2015). Adverse childhood experiences and risk of type 2 diabetes: A systematic review and meta-analysis. Metabolism.

[bib38] Johnson E.I., Easterling B. (2012). Understanding unique effects of parental incarceration on children: Challenges, progress, and recommendations. Journal of Marriage and Family.

[bib39] Jones N.L., Gilman S.E., Cheng T.L., Drury S.S., Hill C.V., Geronimus A.T. (2019). Life course approaches to the causes of health disparities. American Journal of Public Health.

[bib40] Khlat M., Jusot F., Ville I. (2009). Social origins, early hardship and obesity: A strong association in women, but not in men?. Social Science & Medicine.

[bib41] Kruttschnitt C. (2013). Gender and crime. Annual Review of Sociology.

[bib42] Le G.T., Deardorff J., Lahiff M., Harley K.G. (2019). Intergenerational associations between parental incarceration and children's sexual risk taking in young adulthood. Journal of Adolescent Health.

[bib43] Lee R., Fang X., Luo F. (2013). The impact of parental incarceration on the physical and mental health of young adults. Pediatrics.

[bib44] Lee H., Harris K.M., Gordon-Larsen P. (2009). Life course perspectives on the links between poverty and obesity during the transition to young adulthood. Population Research and Policy Review.

[bib45] Lee H., Wildeman C., Wang E.A., Matusko N., Jackson J.S. (2014). A heavy burden: The cardiovascular health consequences of having a family member incarcerated. American Journal of Public Health.

[bib46] Mamun A.A., O'Callaghan M.J., Cramb S.M., Najman J.M., Williams G.M., Bor W. (2009). Childhood behavioral problems predict young adults' BMI and obesity: Evidence from a birth cohort stud. Obesity.

[bib47] Massoglia M., Pridemore W.A. (2015). Incarceration and health. Annual Review of Sociology.

[bib48] Massoglia M., Remster B. (2019). Linkages between incarceration and health. Public Health Reports.

[bib49] Mendez D.D., Thorpe R.J., Amutah N., Davis E.M., Walker R.E., Chapple-McGruder T., Bodnar L. (2016). Neighborhood racial composition and poverty in association with pre-pregnancy weight and gestational weight gain. SSM - Population Health.

[bib50] Moffitt T.E., Caspi A., Harrington H., Milne B.J. (2002). Males on the life-course-persistent and adolescence-limited antisocial pathways: Follow-up at age 26 years. Development and Psychopathology.

[bib51] Murray J., Bijleveld C.C., Farrington D.P., Loeber R. (2014).

[bib52] Newton S., Braithwaite D., Akinyemiju T.F. (2017). Socio-economic status over the life course and obesity: Systematic review and meta-analysis. PLoS One.

[bib53] Pérez-Escamilla R., Kac G. (2013). Childhood obesity prevention: A life-course framework. International Journal of Obesity Supplements.

[bib54] Roettger M.E., Boardman J.D. (2012). Parental imprisonment and gender-based risks for increased BMI: Evidence from a longitudinal study of adolescents and young adults in the US. American Journal of Epidemiology.

[bib55] Roettger M.E., Boardman J.D., Harris K.M., Guo G. (2016). The association between the MAOA 2R genotype and delinquency over time among men: The interactive role of parental closeness and parental incarceration. Criminal Justice and Behavior.

[bib56] Roettger M.E., Dennison S. (2018). Interrupting intergenerational offending in the context of America's social disaster of mass imprisonment. American Behavioral Scientist.

[bib57] Roettger M.E., Houle B. (2021). Assessing the relationship between parental imprisonment in childhood and risk of sexually transmitted infections: A cohort study of US adults in early adulthood. BMJ Open.

[bib58] Roettger M.E., Houle B., Najman J., McGee T.R. (2022). Parental imprisonment as a risk factor for cardiovascular and metabolic disease in adolescent and adult offspring: A prospective Australian birth cohort study. SSM - Population Health.

[bib59] Roettger M.E., Swisher R.R. (2011). Associations of fathers' history of incarceration with sons'delinquency and arrest among black, white, and hispanic males in the United States. Criminology.

[bib60] Semenza D.C., Isom Scott D.A., Grosholz J.M., Jackson D.B. (2020). Disentangling the health-crime relationship among adults: The role of healthcare access and health behaviors. Social Science & Medicine.

[bib61] Smith D.T., Mouzon D.M., Elliott M. (2018). Reviewing the assumptions about men's mental health: An exploration of the gender binary. American Journal of Men's Health.

[bib62] Spatola C.A., Fagnani C., Pesenti-Gritti P., Ogliari A., Stazi M.-A., Battaglia M. (2007). A general population twin study of the CBCL/6-18 DSM-oriented scales. Journal of the American Academy of Child & Adolescent Psychiatry.

[bib63] Su S., Jimenez M.P., Roberts C.T., Loucks E.B. (2015). The role of adverse childhood experiences in cardiovascular disease risk: A review with emphasis on plausible mechanisms. Current Cardiology Reports.

[bib64] Swisher R.R., Roettger M.E. (2012). Father's incarceration and youth delinquency and depression: Examining differences by race and ethnicity. Journal of Research on Adolescence.

[bib65] Sykes B.L., Pettit B. (2014). Mass incarceration, family complexity, and the reproduction of childhood disadvantage. The Annals of the American Academy of Political and Social Science.

[bib66] Testa A., Jackson D.B., Vaughn M.G., Bello J.K. (2019). Incarceration as a unique social stressor during pregnancy: Implications for maternal and newborn health. Social Science & Medicine.

[bib67] Testa A., Semenza D. (2020). Criminal offending and health over the life-course: A dual-trajectory approach. Journal of Criminal Justice.

[bib68] Trotter R.T., Lininger M.R., Camplain R., Fofanov V.Y., Camplain C., Baldwin J.A. (2018). A survey of health disparities, social determinants of health, and converging morbidities in a county jail: A cultural-ecological assessment of health conditions in jail populations. International Journal of Environmental Research and Public Health.

[bib69] Turney K. (2014). Stress proliferation across generations? Examining the relationship between parental incarceration and childhood health. Journal of Health and Social Behavior.

[bib70] Turney K., Wildeman C. (2015). Detrimental for some? Heterogeneous effects of maternal incarceration on child wellbeing. Criminology & Public Policy.

[bib71] Van De Weijer S.G., Smallbone H.S., Bouwman V. (2018). Parental imprisonment and premature mortality in adulthood. Journal of Developmental and Life-Course Criminology.

[bib72] van de Weijer S.G., Besemer K.L., Dennison S.M. (2021).

[bib73] Western B. (2006).

[bib74] Whitaker K.L., Jarvis M.J., Beeken R.J., Boniface D., Wardle J. (2010). Comparing maternal and paternal intergenerational transmission of obesity risk in a large population-based sample. The American Journal of Clinical Nutrition.

[bib75] Wildeman C. (2012). Imprisonment and infant mortality. Social Problems.

[bib76] Wildeman C. (2020). The intergenerational transmission of criminal justice contact. Annual Review of Criminology.

[bib77] Wildeman c., Andersen S.H. (2017). Paternal incarceration and children's risk of being charged by early adulthood: Evidence from a Danish policy shock. Criminology.

[bib78] Wildeman C., Andersen S.H., Lee H., Karlson K.B. (2014). Parental incarceration and child mortality in Denmark. American Journal of Public Health.

[bib79] Wildeman C., Goldman A.W., Turney K. (2018). Parental incarceration and child health in the United States. Epidemiologic Reviews.

[bib80] Wildeman C., Lee H. (2021). Women's health in the era of mass incarceration. Annual Review of Sociology.

[bib81] Wildeman C., Wang E.A. (2017). Mass incarceration, public health, and widening inequality in the USA. The Lancet.

[bib82] Winegar R.K., Lipschitz D.S. (1999). Agreement between hospitalized adolescents' self-reports of maltreatment and witnessed home violence and clinician reports and medical records. Comprehensive Psychiatry.

[bib83] Wiss D.A., Brewerton T.D. (2020). Adverse childhood experiences and adult obesity: A systematic review of plausible mechanisms and meta-analysis of cross-sectional studies. Physiology & Behavior.

[bib84] Yang Y.C., Walsh C.E., Johnson M.P., Belsky D.W., Reason M., Curran P., Aiello A.E., Chanti-Ketterl M., Harris K.M. (2021). Life-course trajectories of body mass index from adolescence to old age: Racial and educational disparities. Proceedings of the National Academy of Sciences.

[bib85] Yu Z., Han S., Zhu G., Zhu C., Wang X., Cao X., Guo X. (2011). Birth weight and subsequent risk of obesity: A systematic review and meta‐analysis. Obesity Reviews.

